# Our Birth

**Published:** 1891-01

**Authors:** 


### OUR BIRTH.

"Our birth is but a sleep and a forgetting :
The soul that rises with us, our life's star,
Hath had elsewhere its setting,
And cometh from afar ;
Not in entire forgetfulness,
And not in utter nakedness,
But trailing clouds of glory do we come
From God, who is our home :"

EXTRACT FROM WORDSWORTH'S INTIMATIONS OF IMMORTALITY

